# Blood-derived macrophages prone to accumulate lysosomal lipids trigger oxLDL-dependent murine hepatic inflammation

**DOI:** 10.1038/s41598-017-13058-z

**Published:** 2017-10-02

**Authors:** Tom Houben, Yvonne Oligschlaeger, Albert V. Bitorina, Tim Hendrikx, Sofie M. A. Walenbergh, Marie-Hélène Lenders, Marion J. J. Gijbels, Fons Verheyen, Dieter Lütjohann, Marten H. Hofker, Christoph J. Binder, Ronit Shiri-Sverdlov

**Affiliations:** 1 Departments of Molecular Genetics, Molecular Cell Biology and Electron Microscopy, School of Nutrition and Translational Research in Metabolism (NUTRIM), University of Maastricht; Universiteitssingel 50, ER 6229 ER, Maastricht, The Netherlands; 2 Institute of Clinical Chemistry and Clinical Pharmacology, University of Bonn; Sigmund-Freud-Str. 25, D-53127 Bonn, Germany; 3 Department of Pathology and Medical Biology, Molecular Genetics, Medical Biology Section, University of Groningen, University Medical Center Groningen; Hanzeplein 1, 9713 GZ Groningen, The Netherlands; 4 Department of Laboratory Medicine, Medical University of Vienna; Spitalgasse 23, 1090 Vienna, Austria; 5 Center for Molecular Medicine (CeMM), Austrian Academy of Sciences; Lazarettgasse 14, A-1090 Vienna, Austria

## Abstract

Despite the consistent rise of non-alcoholic steatohepatitis (NASH) worldwide, the mechanisms that govern the inflammatory aspect of this disease remain unknown. Previous research showed an association between hepatic inflammation and lysosomal lipid accumulation in blood-derived hepatic macrophages. Additionally, *in vitro* findings indicated that lipids, specifically derived from the oxidized low-density lipoprotein (oxLDL) particle, are resistant to removal from lysosomes. On this basis, we investigated whether lysosomal lipid accumulation in blood-derived hepatic macrophages is causally linked to hepatic inflammation and assessed to what extent increasing anti-oxLDL IgM autoantibodies can affect this mechanism. By creating a proof-of-concept mouse model, we demonstrate a causal role for lysosomal lipids in blood-derived hepatic macrophages in mediating hepatic inflammation and initiation of fibrosis. Furthermore, our findings show that increasing anti-oxLDL IgM autoantibody levels reduces inflammation. Hence, therapies aimed at improving lipid-induced lysosomal dysfunction and blocking oxLDL-formation deserve further investigation in the context of NASH.

## Introduction

Analogous to the steep rise of obesity and diabetes, the prevalence of non-alcoholic fatty liver disease (NAFLD) is currently estimated at 25% to 45% in the general population^1,2^. NAFLD encompasses a spectrum of liver diseases that are histologically categorized in nonalcoholic fatty liver (NAFL) and non-alcoholic steatohepatitis (NASH)^3^. Whereas hepatic steatosis without hepatic injury is referred to as NAFL, NASH is defined by a conjunction of steatosis and inflammation, which presents with or without fibrosis^3^. Though the development of inflammation paves the way for advanced liver diseases, the mechanisms underlying the hepatic inflammatory response are largely unknown. As this lack of mechanistic understanding is a key antecedent for the lack of well-defined effective therapies, it is of utmost importance to improve the knowledge regarding the mechanisms triggering hepatic inflammation.

Previous research from our group indicated an association between hepatic inflammation and lysosomal lipid accumulation inside resident Kupffer cells (KCs) as well as in blood-derived macrophages^4–6^. This relation was confirmed by the presence of cholesterol-accumulating KCs in livers of NASH patients, bolstering the notion of lysosomal lipid storage in hepatic macrophages as a potential mechanism for NASH^7^. Also, it has been shown that incubating macrophages with oxLDL *in vitro* results in the lysosomal accumulation of cholesteryl esters (CEs) and free cholesterol, suggesting a direct role for oxLDL in mediating lysosomal lipid-induced inflammation^8,9^. Of note, excessive accumulation of lipids in the lysosomal compartment of cells also occurs in the context of Niemann-Pick type C1 (NPC1) disease. Characterized by hepatosplenomegaly, foam cell formation and hepatic inflammation, NPC1 patients exhibit features resembling NASH^10^. Therefore, it becomes evident that the pathology of lysosomal lipid accumulation is not limited to lysosomal lipid storage diseases such as NPC1, but also plays a role in other lipid-associated inflammatory diseases such as NASH.

Previously, it has been demonstrated that oxLDL and the bacterium *Streptococcus pneumoniae* exhibit molecular mimicry for the phosphorylcholine (PC) epitope, a major target for naturally occurring immunoglobulin M (IgM) antibodies. Immunizing mice with heat-killed *Streptococcus pneumoniae* has been shown to increase anti-oxLDL IgM autoantibodies and to reduce atherosclerotic lesion formation^11^ and hepatic inflammation^5^ in low-density lipoprotein receptor knockout (*Ldlr* ^−/−^) mice. Unlike acetylated LDL (acLDL) and native LDL, injection of oxLDL in hyperlipidemic mice showed increased lysosomal lipid accumulation in hepatic macrophages, corroborating the detrimental effect of oxLDL in promoting inflammation by disturbing the physiology of the lysosome^6^. Altogether, several studies indicate that accumulation of oxLDL and associated lipids in lysosomes of macrophages is associated with increased inflammation. However, none of these studies provide specific evidence that lysosomal storage of lipids in hepatic macrophages can be a mechanistic trigger for inflammation in NASH. Moreover, to what extent oxLDL contributes to lysosomal lipid accumulation-induced hepatic inflammation has to our knowledge never been investigated.

Here, we investigated whether lysosomal lipid accumulation in blood-derived hepatic macrophages is a mechanistic trigger for hepatic inflammation and assessed to what extent anti-oxLDL IgM autoantibodies can affect this mechanism. For this purpose, lysosomal lipid accumulation in blood-derived hepatic macrophages was generated by transplanting bone marrow of Npc1-mutant (*Npc1* ^*mut*^) or wildtype (*Npc1* ^*wt*^) mice into *Ldlr* ^−/−^ mice on a high-fat, high-cholesterol (HFC) diet for 12 weeks. To investigate the specific contribution of oxLDL on lysosomal lipid-induced hepatic inflammation, mice were immunized with heat-killed *Streptococcus pneumoniae* ^5,11^. This study describes lysosomal lipid accumulation in blood-derived hepatic macrophages as a novel mechanism that triggers hepatic inflammation. Moreover, our findings suggest a role for oxLDL in mediating lysosomal lipid-induced hepatic inflammation.

## Results

### Lysosomal lipid accumulation in blood-derived hepatic macrophages results in a severe hepatic pathological phenotype

To ensure successful bone marrow replacement, bone marrow efficiency was assessed. Transplantation of both *Npc1* ^wt^ and *Npc1* ^*mut*^ bone marrow approximated an efficiency of 90% (Supplementary Table S1), proving the bone marrow transplantation successful.

**Figure 1:**
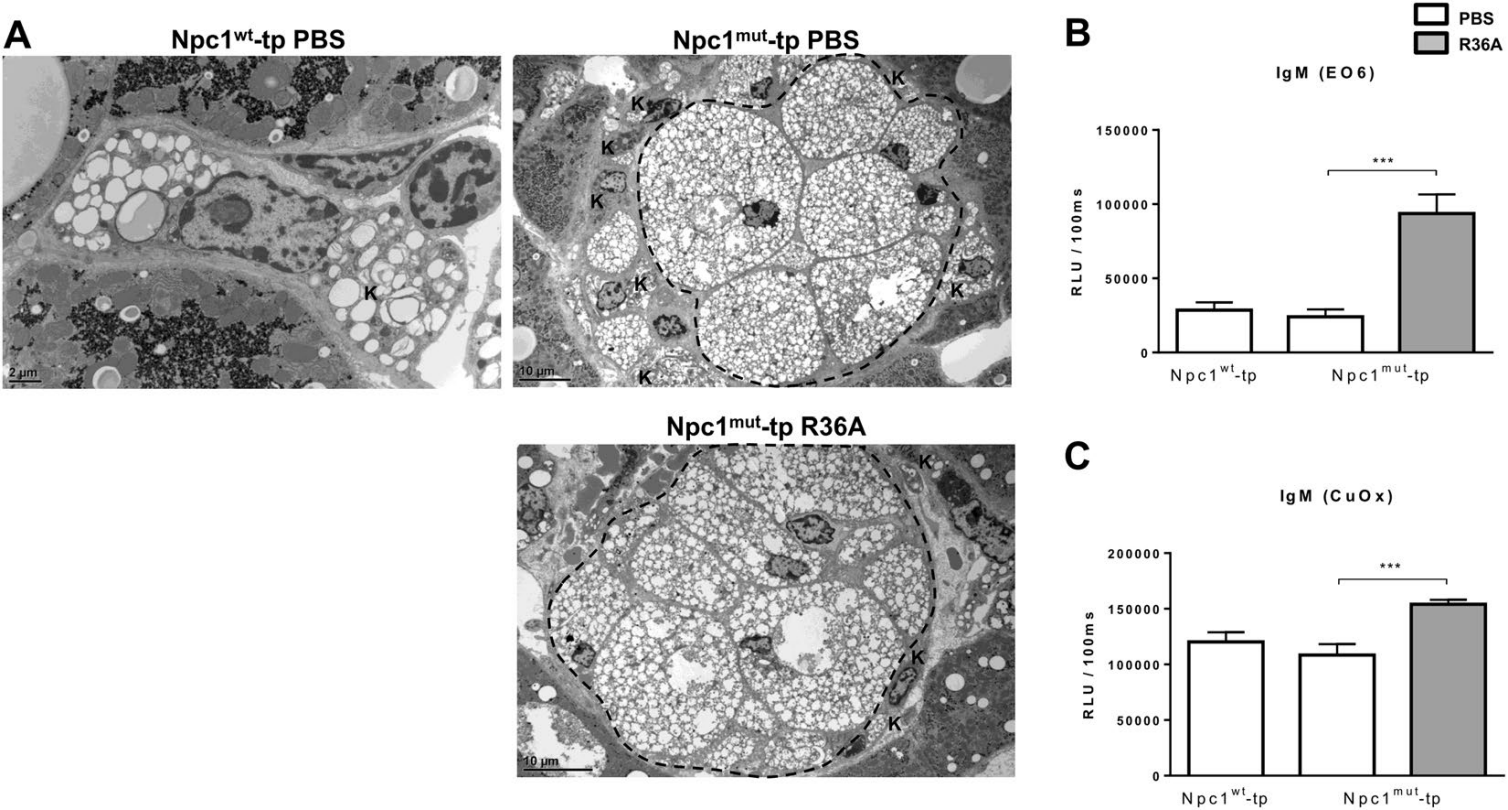
Hepatic phenotype of *Npc1* ^*w*t^-tp and *Npc1* ^*mut*^-tp mice and IgM autoantibody titers in plasma. (**A**) Representative electron microscopy pictures of resident (Kupffer cell) and bone marrow-derived macrophages of *Npc1* ^*wt*^-tp (scale bar 2 μm) and control or immunized *Npc1* ^*mut*^-tp mice (scale bar 10 μm). Area within the dashed line: *Npc1* ^*mut*^ granuloma; K: Kupffer cell. (**B**,**C**) IgM EO6 antibodies (**B**) and IgM antibodies to copper-oxidized (CuOx)LDL (**C**) were measured in plasma of mice with or without immunization at a dilution of 1:100. Data are expressed as relative light units (RLU)/100 ms. n = 10–11 mice/group. Asterisks indicate significant difference from non-immunized *Npc1* ^*mut*^-tp mice by use of two-tailed unpaired *t* test. ***p < 0.001. All error bars are SEM.

Furthermore, to confirm the successful transplantation at the microscopic level, hepatic tissues were subjected to electron microscopy analysis. As pointed out by the lower magnification, livers from *Npc1* ^*mut*^-tp mice demonstrated dense clusters of macrophages which resembled granuloma-like structures. These structures were absent in *Npc1* ^*wt*^-tp mice, indicating that these structures are related to the NPC1 mutation (Fig. 1A). Also, the majority of resident KCs were located adjacent to the large granulomas, containing various numbers of small lipid inclusions (resembling cholesterol crystals (Supplementary Fig. S1)). No detectable differences in phenotype between non-immunized and immunized *Npc1* ^*mut*^-tp mice were observed.

Relevantly, these granuloma structures were also visible at higher microscopic magnification by H&E staining (Supplementary Fig. S2). Also, relative liver and spleen weights were dramatically increased in mice transplanted with *Npc1* ^*mut*^ bone marrow compared to *Npc1* ^*wt*^-tp mice, confirming the severe pathological phenotype (Supplementary Fig. S2). After immunization, both relative liver and spleen weight decreased in immunized *Npc1* ^*mut*^-tp compared to control-treated *Npc1* ^*mut*^-tp, suggesting that inhibition of oxLDL uptake by macrophages ameliorates the pathological phenotype.

### Increased anti-oxLDL IgM autoantibody titers after heat-killed pneumococci immunization

To determine whether immunization with heat-killed *Streptococcus pneumoniae* was performed successfully, IgM autoantibody levels were measured in the plasma. Immunization with heat-killed pneumococci resulted in an increase of plasma IgM antibodies of the EO6/T15 idiotype (Fig. 1B), which bind oxLDL by specifically recognizing the phosphorylcholine epitope^12^. In line, increased IgM antibodies against copper-oxidized LDL (Cu-oxLDL) were detected in immunized mice compared to control mice (Fig. 1C). Thus, immunization with heat-killed pneumococci induced a modest anti-oxLDL IgM autoantibody production in *Npc1* ^*mut*^-tp mice, confirming successfulness of the immunization.

### Disturbances in lipid metabolism are partly restored after elevation of anti-oxLDL IgM autoantibody levels

**Figure 2:**
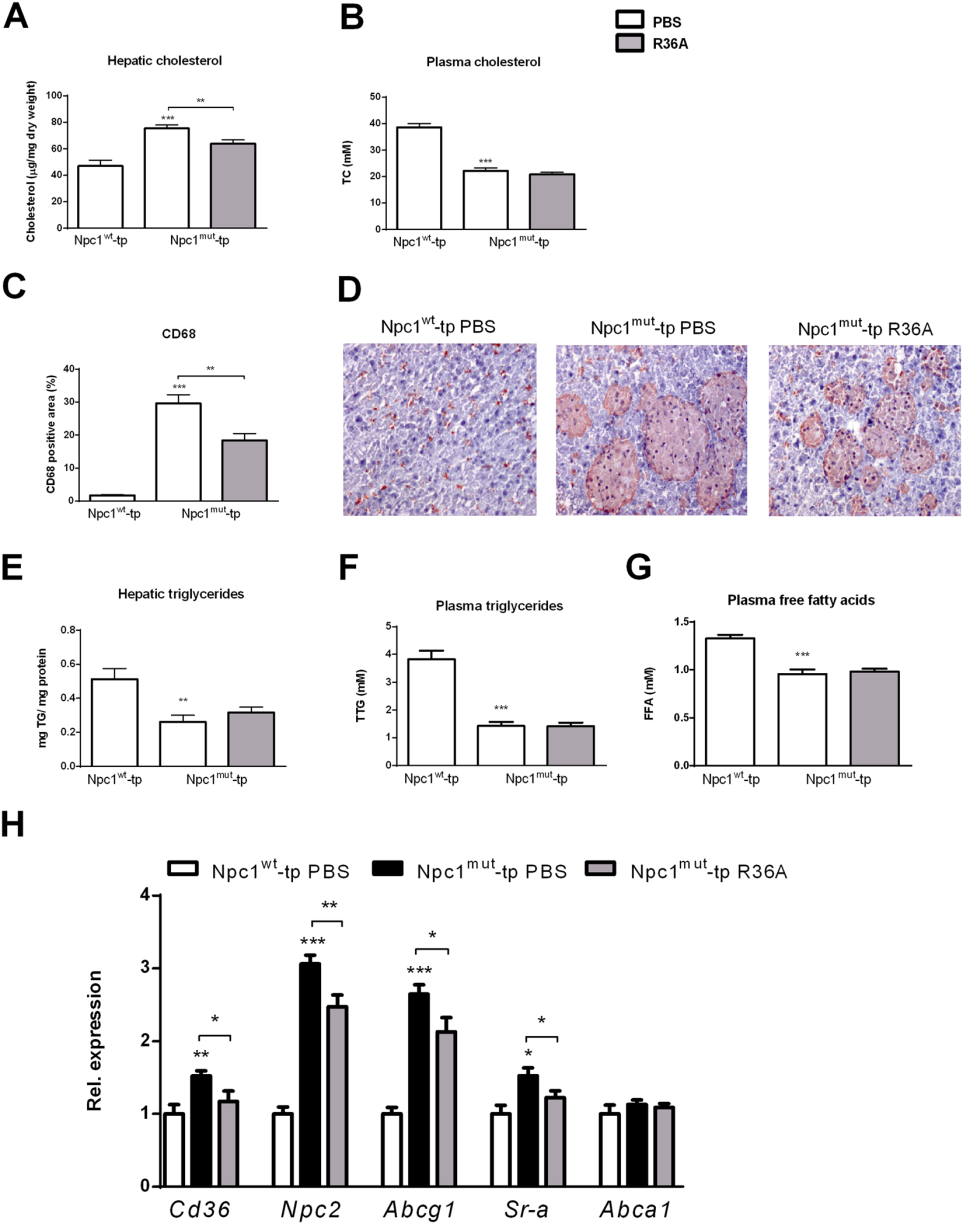
Lipid parameters. (**A**,**B**) Hepatic and plasma cholesterol levels of *Npc1* ^*wt*^-tp and control-treated or immunized *Npc1* ^*mut*^-tp mice on HFC diet. (**C**) Quantification of CD68 staining by measuring CD68 positive area. (**D**) Representative histological pictures of the CD68 staining (200x magnification). (**E**,**F**) Hepatic and plasma triglyceride levels. (**G**) Plasma free fatty acids. (**H**) Hepatic gene expression analysis of *Cd36*, *Npc2* and *Abcg1, Sr-a* and *Abca1*. n = 9–11 mice/group. Gene expression data are set relative to *Npc1* ^*w*t^-tp mice. Asterisks indicate significant difference from non-immunized *Npc1* ^*wt*^-tp and *Npc1* ^*mut*^-tp mice by use of two-tailed unpaired *t* test. *, ** and *** indicate *p* < 0.05, 0.01 and 0.001 resp. All error bars are SEM. TC, total cholesterol; TTG, total triglycerides.

To determine the effect of lysosomal lipid accumulation in hepatic macrophages on lipid metabolism, we examined cholesterol and triglyceride levels in liver and plasma. Whereas hepatic cholesterol levels were elevated in *Npc1* ^*mut*^-tp mice compared to *Npc1* ^*w*t^-tp mice, immunization of *Npc1* ^*mut*^-tp mice decreased hepatic cholesterol, indicating improved hepatic cholesterol metabolism upon immunization (Fig. 2A). In contrast, plasma cholesterol levels reduced by almost 50% in *Npc1* ^*mut*^-tp mice compared to *Npc1* ^*wt*^-tp mice, but did not differ between immunized and non-immunized *Npc1* ^*mut*^-tp mice (Fig. 2B). Detailed investigation of the size of the hepatic granulomas by means of CD68 staining confirmed the presence of granulomas in *Npc1* ^*mut*^-tp mice, while being absent in *Npc1* ^*wt*^-tp mice (Fig. 2C,D). Additionally, immunizing *Npc1* ^*mut*^-tp mice with heat-killed pneumococci resulted in a strong decrease of granuloma size, suggesting oxLDL as an important compound in disturbing cholesterol metabolism in this model (Fig. 2C,D).

Furthermore, hepatic triglyceride levels were decreased in *Npc1* ^*mut*^-tp mice compared to *Npc1* ^*wt*^-tp mice and remained similar between immunized and control-treated *Npc1* ^*mut*^-tp mice (Fig. 2E). Analogous to hepatic triglycerides, similar trends were observed in plasma triglyceride and free fatty acid levels (Fig. 2F,G).

To further define the influences on hepatic lipid metabolism, hepatic expression of genes involved in lipid homeostasis was examined. Compared to *Npc1* ^*wt*^-tp mice, *Npc1* ^*mut*^-tp mice showed increased gene expression levels of Cluster of differentiation 36 (*Cd36*), Scavenger receptor A (*Sr-a*), Niemann-Pick type C2 (*Npc2*) and ATP-binding cassette transporter G1 (*Abcg1*), confirming disturbance of lipid metabolism (Fig. 2H). Furthermore, though no differences were observed in gene expression levels of the ATP-binding cassette transporter A1 (*Abca1*) (Fig. 2H), expression levels of all other markers were reduced upon R36A immunization of *Npc1* ^*mut*^-tp mice, strengthening the importance of oxLDL to the disturbances in lipid metabolism in this model. Altogether, these results imply an important contribution of oxLDL to lysosomal lipid-induced disturbances in lipid metabolism.

### OxLDL contributes to lysosomal lipid-induced hepatic inflammation

**Figure 3:**
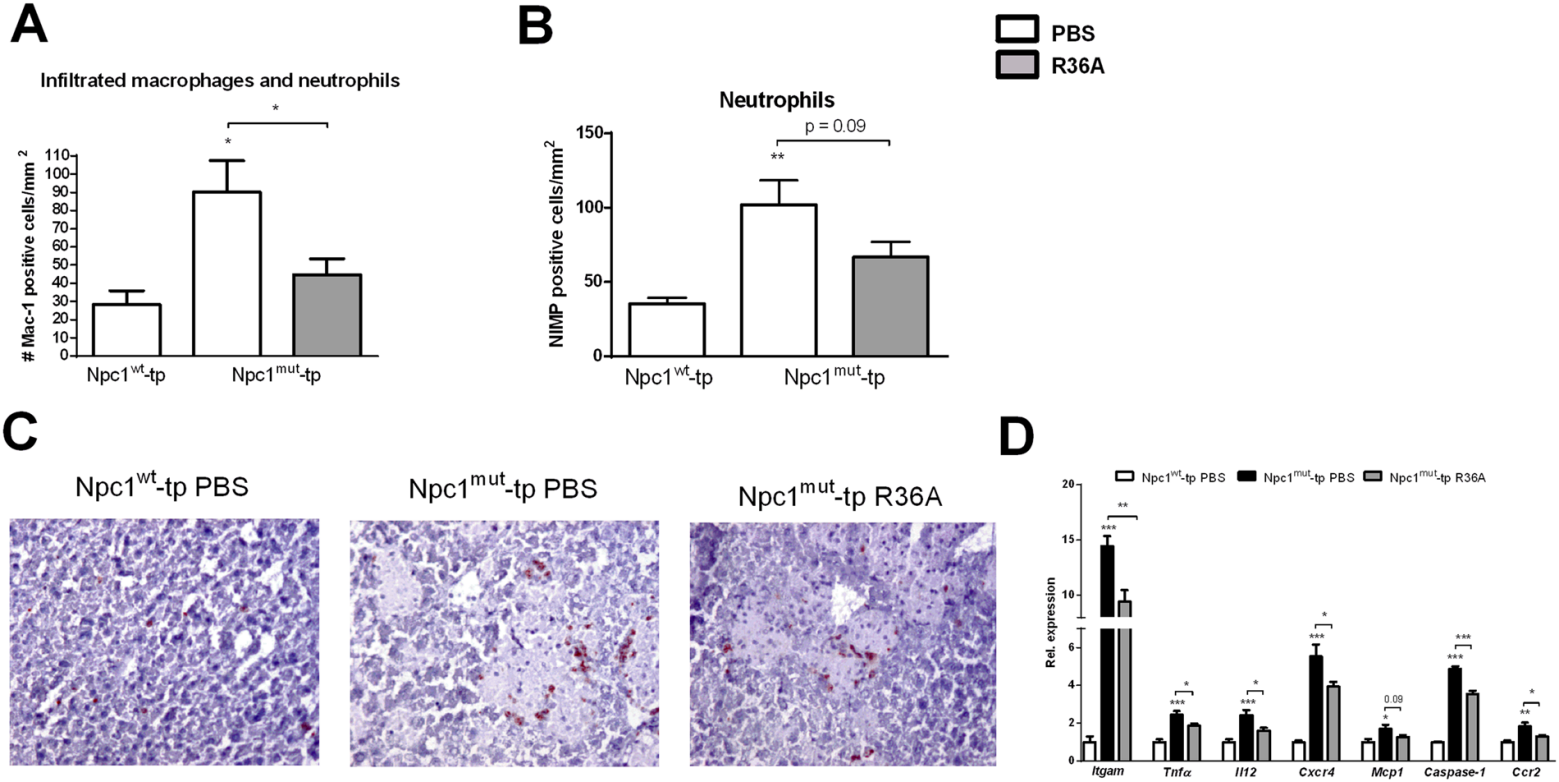
Parameters of hepatic inflammation. (**A**,**B**) Liver sections were stained for both infiltrating macrophages and neutrophils (Mac-1) and neutrophils solely (NIMP) and counted. (**C**) Representative images of the Mac-1 staining (magnification × 200) after HFC feeding of *Npc1* ^*w*t^-tp and control-treated or immunized *Npc1* ^*mut*^-tp mice for 12 weeks. (**D**) Hepatic gene expression analysis of *Itgam, Tnfα, Il12, Cxcr4, Mcp1, Caspase-1* and *Ccr2*. Gene expression data were set relative to *Npc1* ^*wt*^-tp mice. n = 9–11 mice/group. Asterisks indicate significant difference from non-immunized *Npc1* ^*wt*^-tp and *Npc1* ^*mut*^-tp mice by use of two-tailed unpaired *t* test. *p < 0.05; **p < 0.01; ***p < 0.001. All error bars are SEM.

To determine whether lysosomal lipid accumulation in blood-derived macrophages is a trigger for hepatic inflammation, hepatic cryosections were stained for the inflammatory markers Mac-1 (infiltrated macrophages and neutrophils; against Cd11b) and NIMP (neutrophils). Number of cells expressing both inflammatory markers was increased in *Npc1* ^*mut*^-tp mice compared to *Npc1* ^*wt*^-tp mice, supporting our hypothesis that lysosomal lipid accumulation in blood-derived macrophages is a direct trigger for hepatic inflammation (Fig. 3A,B). Additionally, increasing circulating anti-oxLDL IgM autoantibodies reduced Mac-1- and NIMP-positive cell levels (Fig. 3A–C). These inflammatory findings were confirmed by performing hepatic gene expression analysis of the inflammatory markers integrin alpha M (*Itgam*), tumor necrosis factor alpha (*Tnfα*), interleukin 12 (*Il12*), CXC chemokine receptor-4 (*Cxcr4*), monocyte chemoattractant protein 1 (*Mcp1*), *Caspase-1* and CC chemokine receptor-2 (*Ccr2*) (Fig. 3D). Relevantly, while single, just infiltrated macrophages were positive for Mac-1, macrophages present inside the large granulomas of the *Npc1* ^*mut*^-tp group were Mac-1-negative (Fig. 3C). This observation revealed that blood-derived hepatic macrophages lose the Cd11b phenotypic marker over time.

**Figure 4:**
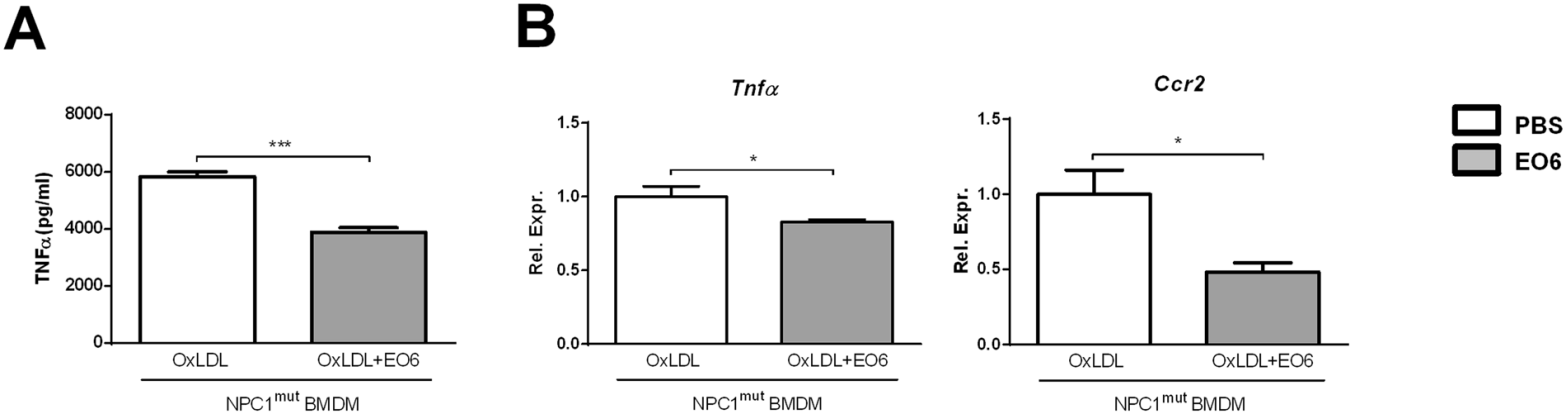
EO6-treatment reduced oxLDL-induced inflammation in *Npc1* ^*mut*^ BMDMs. TNFα protein levels (**A**) and gene expression of inflammatory-related genes *Tnfα* and *Ccr2* (**B**) after oxLDL loading of *Npc1* ^*mut*^ BMDMs in absence or presence of anti-oxLDL EO6 antibodies. All data represent n = 3 (triplicates) for each experimental group. Asterisks indicate significant difference from non-immunized *Npc1* ^*wt*^-tp and *Npc1* ^*mut*^-tp mice by use of two-tailed unpaired *t* test. *p < 0.05; ***p < 0.001. All error bars are SEM.

To confirm the pro-inflammatory properties of oxLDL in bone marrow-derived macrophages (BMDMs), wildtype BMDMs were incubated with oxLDL for 24 hours, followed by 3 hour stimulation with lipopolysaccharide (LPS). Gene expression levels of the pro-inflammatory markers *Tnfα* and *Mcp1* were increased upon oxLDL incubation, confirming the pro-inflammatory effect of oxLDL in BMDMs (Supplementary Fig. S3). Next, to explore the specific contribution of anti-oxLDL IgM autoantibodies to lysosomal lipid-induced inflammation in blood-derived macrophages, we isolated *Npc1* ^*mut*^ BMDMs and stimulated these with oxLDL, in the absence or presence of the anti-oxLDL antibody EO6. In the presence of EO6 antibodies, *Npc1* ^*mut*^ BMDMs stimulated with oxLDL demonstrated reduced inflammation, as indicated by reduced TNFα protein levels and reduced *Tnfα* and *Ccr2* gene expression (Fig. 4).

Combining the *in vivo* and *in vitro* data, these results reveal for the first time that lysosomal lipid accumulation in blood-derived macrophages is a mechanistic trigger for lipid-induced hepatic inflammation. On top, these data identify an essential role for oxLDL in mediating these inflammatory effects.

### Increasing anti-oxLDL IgM autoantibodies attenuates initiation of hepatic fibrosis in mice carrying lysosomal-lipid storing hepatic macrophages

**Figure 5:**
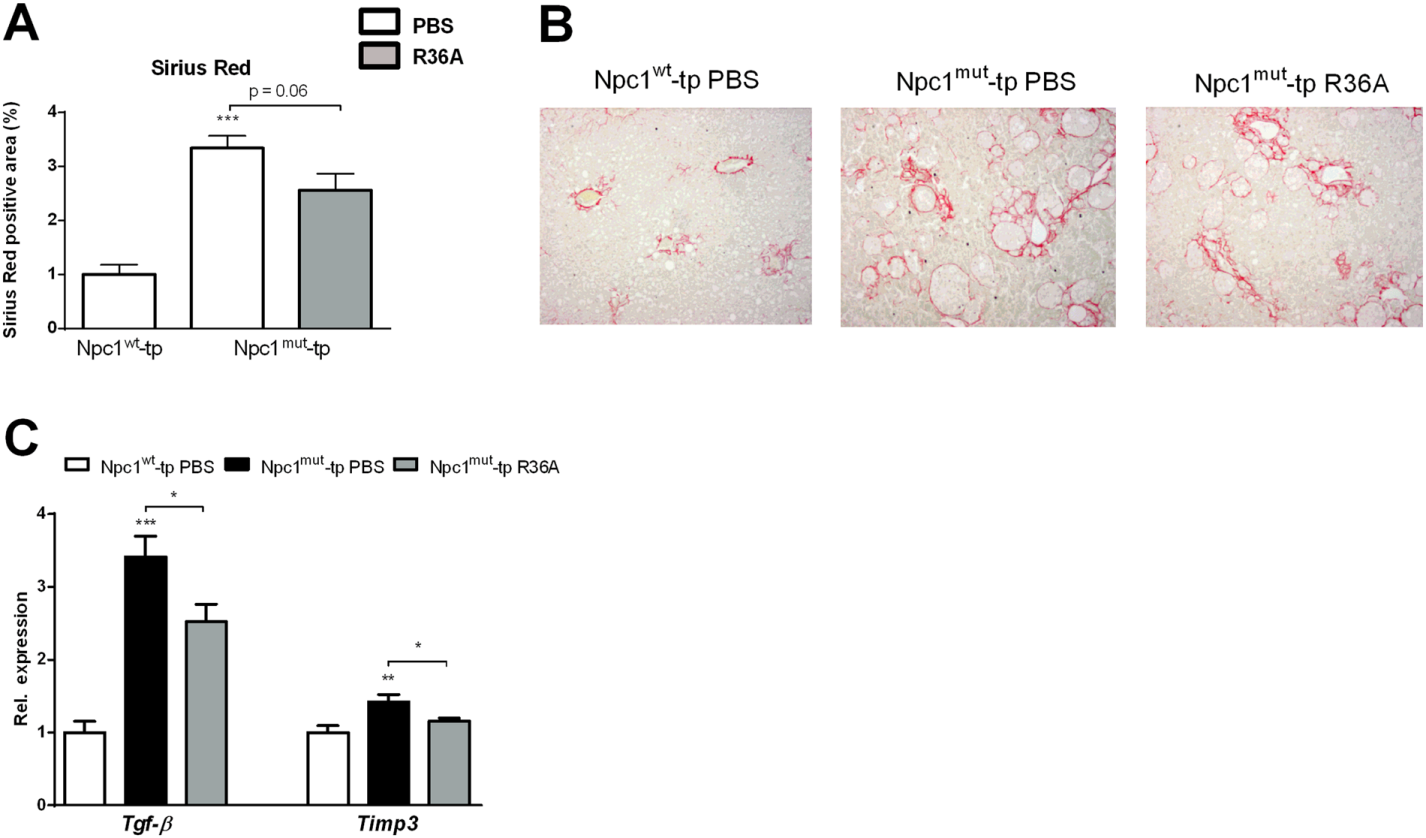
Parameters of hepatic fibrosis. (**A**) Quantification of Sirius Red (collagen) staining. (**B**) Representative pictures of Sirius Red staining (original magnification, 100x) of *Npc1* ^*wt*^-tp mice and *Npc1* ^*mut*^-tp mice with or without immunization on an HFC diet for 3 months. (**C**) Gene expression analysis of the fibrosis markers, *Tgf-β* and *Timp3*. n = 9–11 mice/group. Gene expression data are shown relative to *Npc1* ^*wt*^-tp mice by use of two-tailed unpaired *t* test. *p < 0.05; **p < 0.01; ***p < 0.001. All error bars are SEM.

As hepatic fibrosis is a key symptom of active NASH^13^, we investigated whether the inflammatory trigger of lysosomal lipid accumulation in blood-derived hepatic macrophages can initiate hepatic fibrosis. Hepatic collagen levels, characterized by Sirius Red staining, were elevated in *Npc1* ^*mut*^-tp mice compared to *Npc1* ^*wt*^-tp mice (Fig. 5A,B), as indicated by the increased collagen formation surrounding the granulomas (Fig. 5B). R36A immunization of *Npc1* ^*mut*^-tp mice showed a trend towards a decrease in the level of fibrosis (Fig. 5A). Furthermore, these histological findings were confirmed by gene expression levels of the fibrotic markers, transforming growth factor beta (*Tgf-β*) and tissue inhibitor of metalloproteinase-3 (*Timp3*) (Fig. 5C). Altogether, these data suggest that oxLDL can initiate hepatic fibrosis by contributing to lysosomal lipid accumulation in blood-derived hepatic macrophages.

## Discussion

Currently, the mechanisms underlying NASH are poorly understood, restricting the development of well-defined, effective therapies. Previously, an association was demonstrated between murine hepatic inflammation and lysosomal lipid accumulation in hepatic macrophages^4–6^. Here, we prove that lysosomal lipid accumulation in blood-derived hepatic macrophages is a direct trigger for hepatic inflammation and initiates fibrosis. Moreover, our results show that increasing anti-oxLDL IgM autoantibody levels improves inflammation and lipid metabolism, suggesting that oxLDL plays a key role in mediating hepatic inflammation by promoting lipid accumulation in lysosomes of blood-derived hepatic macrophages. Therefore, this study provides additional insights into the inflammatory mechanisms driving NASH. Therapies aimed at improving lipid-induced lysosomal dysfunction and blocking the formation of oxLDL should therefore be investigated in the future.

Whereas initially viewed as the cell’s degradation center, it has become increasingly clear that the lysosome constitutes a central role in regulating a plethora of physiological processes^14^. Specifically, upon accumulation of macromolecules (including lipids), lysosomes gradually lose their proteolytic and regulatory function, leading to disturbances in processes related to apoptosis, autophagy, calcium homeostasis, protein folding and, above all, metabolism and inflammation^15^. Indeed, severely increased inflammation was observed in NPC1^16^ and Wolman^17^ disease patients, both lysosomal lipid storage disorders (LSDs), thereby linking lipid storage to inflammation. Likewise, previous studies by us and others have demonstrated an association between hepatic inflammation and lysosomal lipid accumulation^5,6,18^. Building on this previous knowledge, we here demonstrate for the first time that lysosomal lipid storage in blood-derived hepatic macrophages can be viewed as an actual trigger for hepatic inflammation and initiates fibrosis, two central features of NASH. Our data also imply that oxLDL contributes to this hepatic inflammatory mechanism. As such, we propose that lysosomal accumulation of oxLDL-derived lipids is a trigger for NASH.

The observation that elevations in anti-oxLDL IgM autoantibody levels could partly protect from the inflammatory phenotype observed in *Npc1* ^*mut*^-tp *Ldlr* ^−/−^ mice suggests that an important fraction of lysosomal lipids in blood-derived hepatic macrophages originates from oxLDL particles. In line, while circulating levels of oxLDL were reported to represent 0.001% of native LDL in healthy individuals^19^, oxLDL levels were shown to increase up to 1.8% in patients with the metabolic syndrome^20^. Relevantly, 7β-hydroxycholesterol and 7-ketocholesterol, the main cholesterol oxidation products present inside oxLDL particles^21,22^, were previously linked to inflammatory processes, suggesting that cholesterol oxidation products contained in the oxLDL particle are responsible for eliciting inflammation^23,24^. Also, oxLDL-induced formation of cholesterol crystals was demonstrated to induce lysosomal membrane permeabilization^25–27^. Lysosomal membrane permeabilization and the subsequent release of lysosomal enzymes into the cytoplasm is a known prerequisite for activation of the inflammasome^28^, increased apoptosis^29^ and of necrotic cell death^30^, three pathways leading to increased hepatic inflammation. In line with these observations, blood-derived hepatic *Npc1* ^*mut*^ macrophages exhibited ruptured membrane structures, suggesting that cholesterol oxidation products induced lysosomal permeabilization in these macrophages resulting in hepatic inflammation. However, more evidence is needed to prove the actual contribution of cholesterol oxidation products to lysosomal permeabilization. Furthermore, to what extent oxLDL levels in our mouse model are comparable to oxLDL levels in NASH patients is not completely clear. Nevertheless, as both NASH patients^31^ and *Npc1* ^*−/–*^ tp *Ldlr* ^−/−^ mice (described by Zhang *et al*.^32^) show increased levels of plasma oxidized lipids, our mouse model can be considered a proper model to investigate the contribution of oxidized lipids to the human NASH situation. Oxidation of lipids were also shown to occur within lysosomes^33^. Therefore, it is likely that the immunized *Npc1* ^*mut*^-tp group still contains internally oxidized lipid products within lysosomes, which contribute to the hepatic inflammatory response.

Besides cholesterol oxidation products, other components present inside or in the surface monolayer of oxLDL particles have also been associated with inflammatory responses. For example, oxidized phopspholipids and their highly reactive degradation product malondialdehyde, two lipid products identified on the surface of the oxLDL particle^34,35^, have been identified in NASH patients^31,36,37^. Furthermore, other lipids such as free cholesterol, cholesteryl esters, proteins and their oxidized derivatives have been identified in oxLDL^24^. Additionally, while next to oxidized lipids also other non-oxidized cholesterol products contribute to the observed pathology, the finding that targeting specifically oxidized LDL leads to significant improvements in liver pathology provides evidence for an essential role for oxLDL in mediating lysosomal lipid-induced hepatic inflammation. Therefore, future research should aim to identify and provide detailed knowledge of the lipids specifically contained in oxLDL particles as this might lead to the identification of novel therapeutic targets for NASH.

Of note, besides oxLDL, also apoptotic cells expose the PC-epitope which is recognized by IgM autoantibodies described in this manuscript^38^. As increased apoptosis has often been associated with increased levels of hepatic inflammation^39,40^, it is possible that elevations in IgM autoantibody levels reduced hepatic inflammation in our model via increased clearance of apoptotic cells, rather than preventing the uptake of oxLDL by hepatic macrophages.

The current study also describes that blood-derived hepatic macrophages, which exhibited lysosomal lipid accumulation due to a NPC1 mutation, lose the CD11b phenotypic marker (indicative for hematopoietic origin^41^) and gain the CD68 phenotypic marker (indicative for resident KCs^41^) after infiltration in the liver, suggesting that blood-derived macrophages have phenotypically switched into resident KCs. Additionally, embryonically-derived resident KCs displayed intracellular cholesterol crystal formation and engulfed the granulomas (which appeared to be the result of a clustering of blood-derived hepatic macrophages carrying the NPC1 mutation). Therefore, the inability to eliminate the inflammatory stimulus (which is derived from the granulomas created by blood-derived hepatic macrophages) in the liver is likely due to a combination of dysfunctional blood-derived hepatic macrophages as well as the lack of a functional repertoire of resident embryonically-derived KCs to overcome this insult. Indeed, under inflammatory conditions, blood-derived tissue-resident macrophages were shown to dominate the inflammatory response in tissues^42^. Therefore, the findings of the current study indicate that functional blood-derived hepatic macrophages are essential to overcome lipid challenges in the liver and suggest that improving the lysosomal function in macrophages (from hematopoietic or embryonic origin) can be beneficial for NASH.

Although a bone marrow-specific NPC1 mutation was used in this study, the phenomenon of lysosomal lipid accumulation in hepatic macrophages in NASH patients is not necessarily the result of a deficiency of the *Npc1* gene, but rather a consequence of a prolonged exposure of lipids to the liver. Furthermore, patients suffering from NASH traditionally show increased hepatic levels of cholesterol and triglycerides^2^, while our model only showed elevations in hepatic cholesterol levels. Therefore, the mouse model described in this study should be considered a proof-of-concept mouse model for the involvement of lysosomal lipid storage in blood-derived hepatic macrophages to hepatic inflammation and fibrosis rather than a mouse model exactly mimicking the human situation of NASH.

## Conclusions

In conclusion, we demonstrate here for the first time that lipid accumulation in lysosomes of blood-derived hepatic macrophages is a key trigger of hepatic inflammation and mediates initiation of fibrosis. Furthermore, increasing anti-oxLDL IgM autoantibody levels ameliorated the pathological phenotype, suggesting a key role for oxLDL in this process. Hence, therapies aimed at improving lipid-induced lysosomal dysfunction and blocking the formation of oxLDL should be further investigated in the context of NASH and might be of relevance for other metabolic inflammatory disorders.

## Methods

### Mice, bone marrow transplantation, immunization, and diet

Niemann-Pick type C1^nih^ mutant (*Npc1* ^*mut*^) mice (a kind gift from Prof. Dr. Lieberman from University of Michigan Medical School) were backcrossed into a C57BL/6 background for more than 10 generations. *Npc1* ^*mut*^ and *Ldlr* ^−/−^ mice were housed under standard conditions and had access to food and water ad libitum. Experiments were performed according to Dutch regulations and approved by the Committee for Animal Welfare of Maastricht University.

To generate myeloid *Npc1* ^*mut*^ deficient *Ldlr* ^−/−^ mice, bone marrow transplantations were performed. Twenty-two week-old female *Ldlr* ^−/−^ mice received antibiotic water containing neomycin (100 mg/l; Gibco, Breda, the Netherlands) and 6*10^4^ U/l polymycin B sulphate (Gibco, Breda, the Netherlands) one week before and four weeks after irradiation. One day before and on the day of the transplantation, *Ldlr* ^−/−^ mice were lethally irradiated with 6 Gray of γ-radiation, thus receiving 12 Gray in total. Lethally irradiated *Ldlr* ^−/−^ mice were then injected with 1*10^7^ bone marrow cells donated from either *Npc1* ^*mut*^ mice or wildtype littermate controls (*Npc1* ^*wt*^). In order to fully ensure bone marrow replacement, mice had a nine week recovery period. After nine weeks of recovery, transplanted (-tp) mice received a high-fed, high-cholesterol (HFC) diet, containing 21% butter and 0.2% cholesterol (diet 1635; Scientific Animal Food and Engineering, Villemoissonsur-Orge, France) for 12 weeks. Five weeks after bone marrow transplantation, mice were divided into three groups. One group received the equivalent of 10^8^ colony-forming units of heat-killed R36A (unencapsulated Streptococcus pneumoniae) emulsified in 200 µl sterile 0.9% NaCl for the primary subcutaneous immunization. Subsequently, two intraperitoneal booster immunizations were administered every two weeks. The two other control groups received an 0.9% NaCl injection only. From the start of the diet, intraperitoneal booster immunizations were administered every three weeks. An overall overview of the experimental set-up is depicted in Supplementary Fig. S4.

Liver tissue was isolated and snap-frozen in liquid nitrogen and stored at −80 °C or fixed in 4% formaldehyde/PBS. The biochemical determination of plasma cholesterol and liver triglyceride levels, electron microscopy, RNA isolation, complementary DNA synthesis, quantitative polymerase chain reaction and auto-antibody titers against anti-oxLDL IgM antibodies are described extensively^4,5,43–46^. Liver cholesterol levels were quantified as described previously^47^.

### Bone marrow-derived macrophages

Bone marrow-derived macrophages (BMDMs) were isolated from the tibiae and femurs of wildtype and *Npc1* ^*mut*^ mice. Cells were cultured in RPMI-1640 (GIBCO Invitrogen, Breda, the Netherlands) with 10% heat-inactivated fetal calf serum (Bodinco B.V. Alkmaar, the Netherlands), penicillin (100 U/ml), streptomycin (100 μg/ml) and L-glutamine 2 mM (all GIBCO Invitrogen, Breda, the Netherlands), supplemented with 20% L929-conditioned medium (LCM) for 8–9 days to generate BMDMs. After attachment, *Npc1* ^*mut*^ macrophages were seeded at 350,000 cells per well in 24-well plates and incubated for 24 h with oxLDL (25 μg/ml; Alfa Aesar: J65591, Wardhill, MA, USA), with or without anti-oxLDL EO6 antibodies (Avanti Polar Lipids, Alabaster, AL, USA). Wildtype macrophages underwent a similar procedure, but did not receive the EO6 antibodies. Then cells were washed and stimulated with lipopolysaccharide (LPS; 100 ng/ml) for 3 h (wildtype cells) or 4 h (*Npc1* ^*mut*^ cells). Finally, supernatant was collected for protein measurements and cells were lysed for mRNA expression analysis.

### Statistical analysis

Data were statistically analyzed by performing two-tailed non-paired *t*-tests using GraphPad Prism, version 6.0 for Windows. Data were expressed as the mean ± SEM and considered significant at *p* < 0.05. *, ** and *** indicate p < 0.05, 0.01 and 0.001 respectively. Additional explanation is provided in Supplementary Information.

## Electronic supplementary material

Supplementary Information
