# Supplementary material for: Blood-derived macrophages prone to accumulate lysosomal lipids trigger oxLDL-dependent murine hepatic inflammation
Source: Sci Rep. 2017 Oct 2;7:12550. doi: 10.1038/s41598-017-13058-z (PMC5624963; doi:10.1038/s41598-017-13058-z)
Supplement: Supplementary file 1 — Supplementary Information [file 41598_2017_13058_MOESM1_ESM.pdf]

Supplementary Information

Blood-derived macrophages prone to accumulate lysosomal lipids trigger oxLDL-dependent murine hepatic inflammation

Tom Houben[1], Yvonne Oligschlaeger[1], Albert V Bitorina[1], Tim Hendrikx[1], Sofie MA Walenbergh[1], Marie-Hélène Lenders[1], Marion JJ Gijbels[1], Fons Verheyen[1], Dieter Lütjohann[2], Marten H Hofker[3], Christoph J Binder[4,5], Ronit Shiri-Sverdlov[1*]

Supplementary Table 1

Bone marrow efficiency of *Npc1*^{wt}- and *Npc1*^{mut}-tp *Ldlr*$^{-/-}$ mice

Genotype	% LDLR
Npc1^{wt}*-tp Ldlr*$^{-/-}$ mice	90.47 ± 6.96
Npc1^{mut}*-tp Ldlr*$^{-/-}$ mice	87.96 ± 2.21

Supplementary Fig. S1

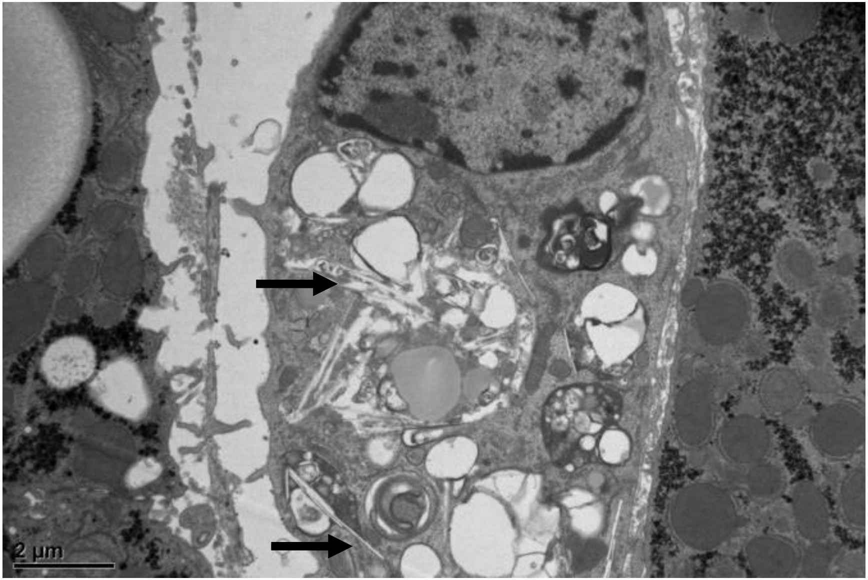

Supplementary Fig. S1: Hepatic phenoytype of *Npc1^{mut}*-tp mice.
The formation of cholesterol crystals was observed in Kupffer cells (indicated by the arrows) that engulfed the granuloma-like structures in *Npc1^{mut}*-tp mice.

Supplementary Fig. S2

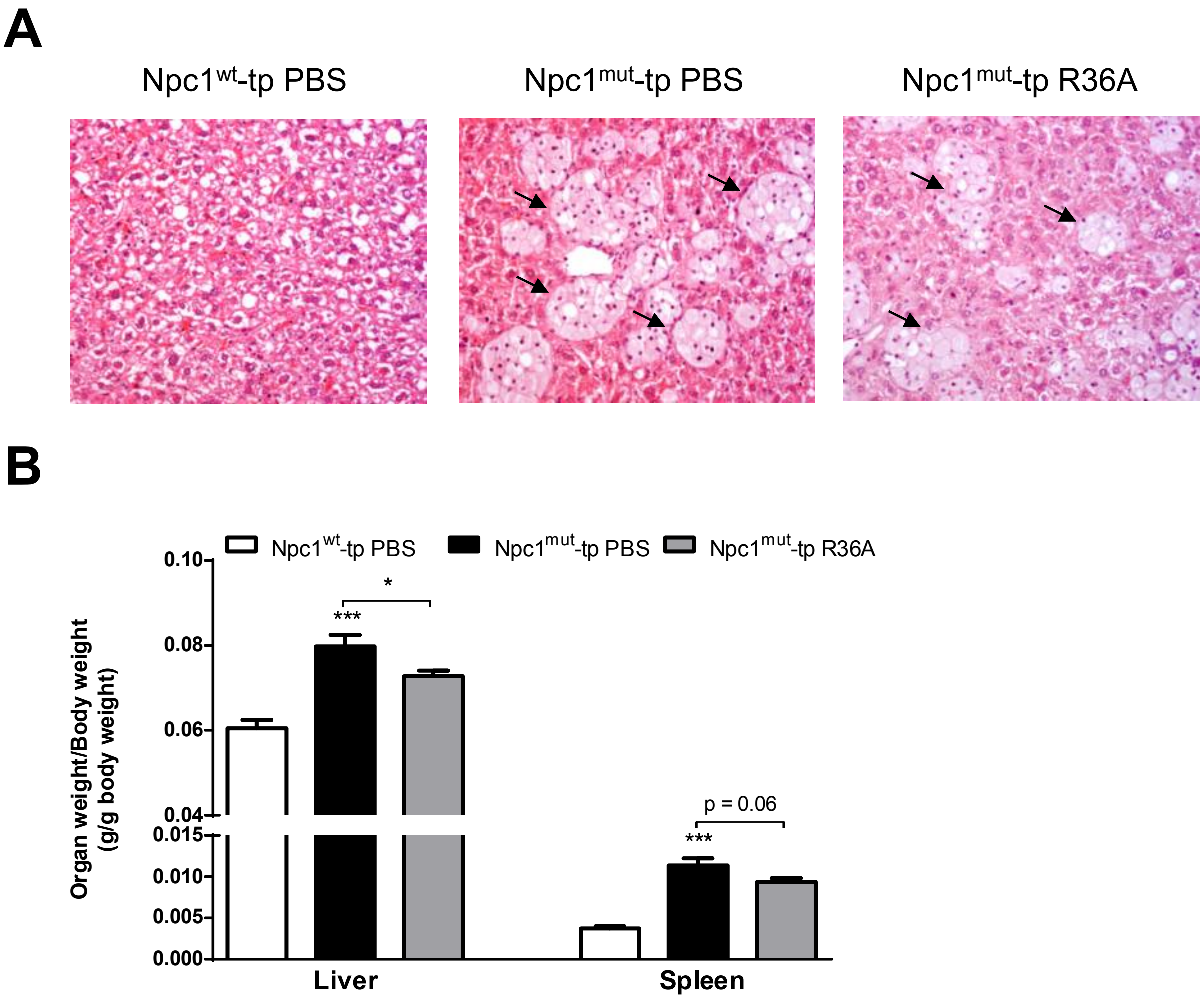

Supplementary Fig. S2: **Hepatosplenic pathological phenotype in *Npc1^{mut}*-tp *Ldlr$^{-/-}$* mice.** (A) General histology of the liver by an HE staining of *Npc1^{wt}*-tp mice and non-immunized and immunized *Npc1^{mut}*-tp mice. Arrows indicate macrophage granulomas. (B) Relative liver and spleen weights. n = 9-11 mice/group. Asterisks indicate significant difference from non-immunized *Npc1^{wt}*-tp and *Npc1^{mut}*-tp mice by use of two-tailed unpaired *t* test. * $p < 0.05$; *** $p < 0.001$. All error bars are SEM. R36A, unencapsulated Streptococcus pneumoniae

Supplementary Fig. S3

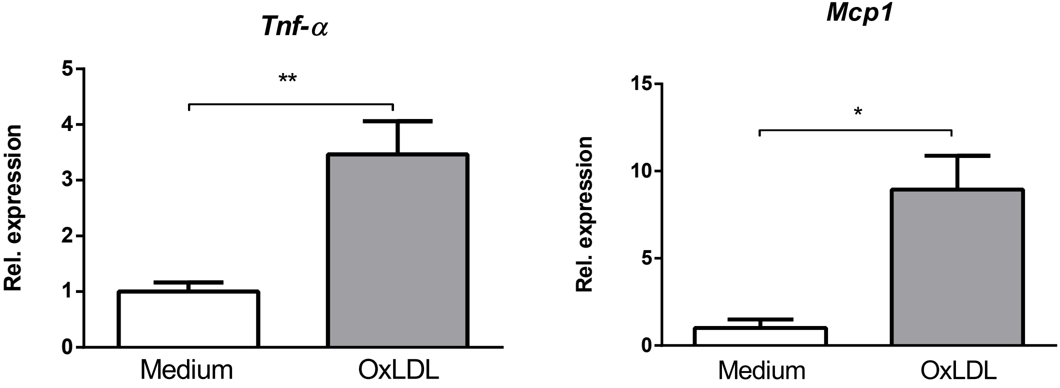

Supplementary Fig. S3: Pro-inflammatory properties of oxLDL in BMDMs.

Gene expression levels of the pro-inflammatory markers *Tnfα* and *Mcp1* after oxLDL loading of wildtype BMDMs. Data are the result of three independent experiments. Asterisks indicate significant difference from control-treated BMDMs by two-tailed unpaired *t* test. * $p < 0.05$; ** $p < 0.01$. All error bars are SEM.

Supplementary Fig. S4

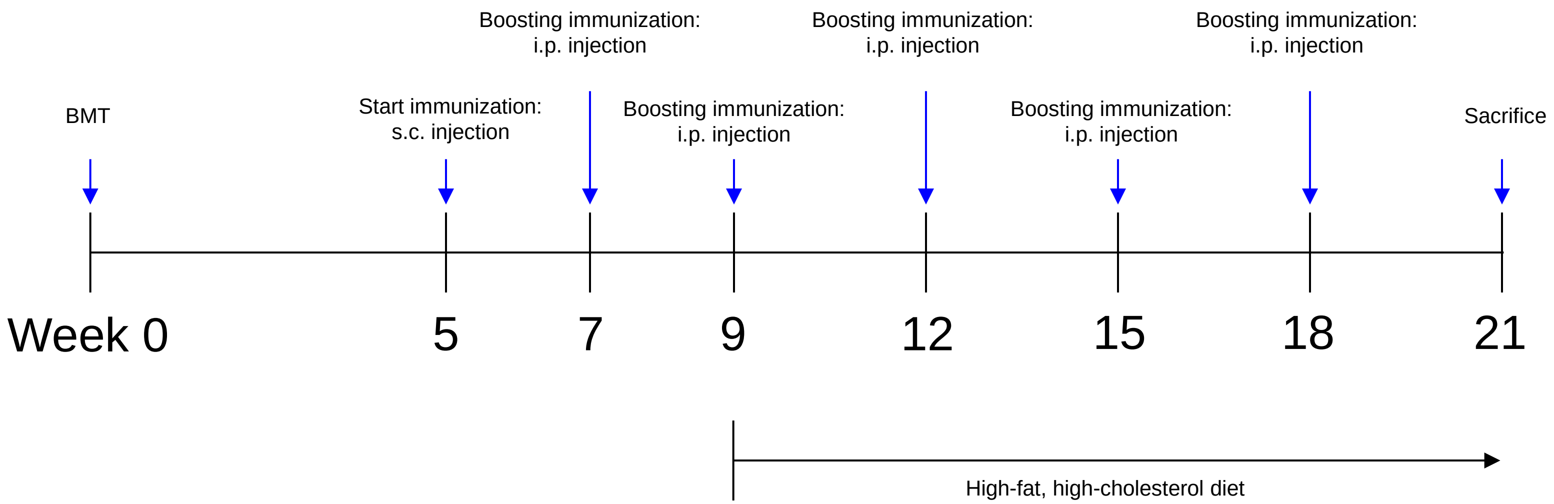

Supplementary Fig. S4: Schematic overview of experimental set-up.

Supplementary Methods

Bone marrow efficiency

In order to determine the chimerism in the transplanted mice, we used donor bone marrow that has an $Ldlr^{WT}$ origin, whereas recipient bone has an $Ldlr^{-/-}$ origin. Genomic DNA was isolated using the PureLink Genomic DNA (K182002; ThermoFisher Scientific). A standard curve was generated by mixing DNA from $Ldlr^{-/-}$ and $Ldlr^{WT}$ bone marrow cells at different ratios. Chimerism was determined by quantifying the amount of $Ldlr^{-/-}$ DNA in samples from 70 µL peripheral blood. To standardize for the amount of input DNA, the non-relevant p50 gene was quantified. Samples were assayed in duplicate on a 7900HT real-time PCR system by using 25 ng DNA, SensiMix^{TM} Sybr & Fluorescein kit (QT615-05, Bioline), according to the manufacturer's instructions.

$Ldlr^{-/-}$-specific primers are forward 5'- GCTGCAACTCATCCATATGCA-3' and reverse 5'-GGAGTTGTTGACCTCGACTCTAGAG-3'. Forward and reverse p50-specific primers are 5'-ACCTGGGAATACTTCATGTGACTAA-3' and 5'-ACACCAGAAGTCCAGGATTATCAG-3', respectively. A standard curve was generated by plotting the mean threshold cycle (Ct) ΔCt (Ct p50 – Ct $Ldlr^{-/-}$) against the logarithm of the percentage $Ldlr^{-/-}$ and calculation of a regression line. Chimerism was calculated from the percentage of $Ldlr^{-/-}$ DNA in the blood samples (representing the remaining recipient bone marrow), determined by applying the mean ΔCt of the sample to the standard curve. The average efficiency of the bone marrow transplantation was approximately 90% (Table S1).

Preparation immunogen

For immunization, the heat-inactivated R36A strain of *Streptococcus pneumoniae* (Birmingham, Alabama) was used, still bearing the PC headgroup epitope similar to oxLDL. Colonies of the R36A strain were harvested at mid log phase after incubation at 37°C on blood agar plates and transferred to Todd-Hewitt plus 0.5% yeast broth. The mid log phase is characterized by an OD value of 0.425 to 0.45 at 600 nm. *S. pneumoniae* was heat-inactivated at 60°C for 30 minutes; afterwards no colonies of this suspension were detected on blood agar plates. For freezer stocks of strain R36A, small aliquots of *S. pneumoniae* at mid log density were harvested and suspended in Todd-Hewitt plus 80% sterile glycerol and stored at −80°C.

Liver histology

Frozen liver sections (7 µm) were fixed in acetone and subsequently blocked for endogenous peroxidase by incubation with 0.25% of 0.03% H_2O_2 for 5 minutes. Primary antibodies used were against infiltrated macrophages and neutrophils (rat-anti-mouse Mac-1 [M1/70]), neutrophils (rat-anti-mouse Ly6-C, clone NIMP-R14) (generous gift from Prof Heeringa, Groningen, The Netherlands) and Kupffer cells (KCs) (rat-anti-mouse CD68, clone FA11) (generous gift from Prof Gordon, Oxford, UK) was applied as color substrate and hematoxylin (4085.9002, Klinipath, Duiven, The Netherlands) for nuclear counterstaining. Sections were enclosed with Faramount aqueous mounting medium (S302580; DAKO, Glostrup, Denmark).

Paraffin-embedded liver sections (4 μm) were stained with Hematoxylin-Eosin (HE; Hematoxilin, 4085.9002; Klinipath, Duiven, The Netherlands; and Eosin, E4382; Sigma-Aldrich) and Sirius red (Direct Red 80, 43665; Sigma-Aldrich). Pictures were taken with a Nikon digital camera DMX1200 and ACT-1 v2.63 software (Nikon Instruments Europe, Amstelveen, The Netherlands).

The CD68 positive area was quantified in 3 microscopical views of the liver by using Adobe Photoshop v. 9.0 in a blinded manner. The number of Mac1- and NIMP1-positive immune cells was counted in 6 microscopical views of each liver section (original magnification, 200×) in a blinded manner and was noted as cells/square millimeter. n = 6-10 mice/group.

The Sirius Red positive area was quantified in 6 microscopical views of the liver by using Adobe Photoshop v. 9.0 in a blinded manner.

Mouse tumor necrosis factor enzyme-linked immunosorbent assay (ELISA)

The mouse tumor necrosis factor ELISA assay was performed on diluted supernatant from stimulated BMM (1:10) according to the manufacturer's instructions (88-7324-88; eBioscience, San Diego, CA, USA).
